# Impact of Endothelial Microparticles on Coagulation, Inflammation, and Angiogenesis in Age-Related Vascular Diseases

**DOI:** 10.1155/2013/734509

**Published:** 2013-10-28

**Authors:** Margaret Markiewicz, Erin Richard, Natalia Marks, Anna Ludwicka-Bradley

**Affiliations:** ^1^Division of Rheumatology and Immunology, Medical University of South Carolina, 114 Doughty Street, STB, Charleston, SC 29425, USA; ^2^Department of Biology, College of Charleston, Rita Liddy Hollings Science Center, Charleston, SC 29424, USA; ^3^Department of Radiology, Maimonides Medical Center, Brooklyn, NY 11219, USA

## Abstract

Endothelial microparticles (EMPs) are complex vesicular structures that originate from plasma membranes of activated or apoptotic endothelial cells. EMPs play a significant role in vascular function by altering the processes of inflammation, coagulation, and angiogenesis, and they are key players in the pathogenesis of several vascular diseases. Circulating EMPs are increased in many age-related vascular diseases such as coronary artery disease, peripheral vascular disease, cerebral ischemia, and congestive heart failure. Their elevation in plasma has been considered as both a biomarker and bioactive effector of vascular damage and a target for vascular diseases. This review focuses on the pleiotropic roles of EMPs and the mechanisms that trigger their formation, particularly the involvement of decreased estrogen levels, thrombin, and PAI-1 as major factors that induce EMPs in age-related vascular diseases.

## 1. Introduction

Vascular diseases are among the most common causes of morbidity and mortality, and both number and severity of morbid vascular conditions increase with age. Regulations of angiogenesis, coagulation, and inflammation are very important issues in vascular biology, both in normal physiology and pathology [1]. It is now well established that disruption of endothelial integrity represents a crucial event in the initiation and development of cardiovascular (CV) diseases. Numerous studies have reported that microparticles (MPs) play an important role in endothelial dysfunction. Endothelial dysfunction occurs when a perturbed homeostatic endothelium disrupts vascular competency resulting in reduced vasodilatation and increased proinflammatory and prothrombotic properties of the vascular network [2]. Recently, MPs originating from various cells have been found to be associated with several vascular related diseases. Moreover, exposed procoagulant phospholipids and specific receptors at the surface of MPs act as biomessengers linking inflammation, coagulation, and angiogenesis [3–5]. 

Although MPs were first described as “cellular debris” that are believed to have no biological significance, recent studies documented that MPs of endothelial and other origins are biological effectors in inflammation, vascular injury, angiogenesis, and thrombosis [6–8]. MPs isolated from granulation tissue are derived from endothelial cells, monocytes, platelets, erythrocytes [9–13], and myofibroblasts [8]. They exchange biological signals and information intercellularly and each kind of MP carries the antigens and receptors of the cells they originated. MPs may transfer part of their components and content to the selected target cells, thus mediating cell activation, phenotypic modification, and reprogramming of cell function [14]. Although 70% to 90% of all circulating MPs in the peripheral blood of healthy individuals are derived from platelets [15], marked elevations of all kinds of MPs have been observed in many vascular diseases. Specifically, endothelium-derived microparticles (EMPs) represent a relatively small (5–15%) but very important subset of all circulating microparticles [16–18]. This number may vary in different cardiovascular and inflammatory diseases [18, 19]. New insights into endothelial dysfunction and alterations in angiogenesis are emerging from studies of vascular microparticles, particularly endothelial microparticles in elderly populations.

Age-related CV diseases are considered a major concern for the elderly. Vascular aging with impairment of endothelial cell function leads to altered angiogenesis, a key factor in the etiology of various cardiovascular disorders. 73% of individuals aged 60–79 have a CV disease, including stroke, hypertension, or heart failure, and at >79 years of age prevalence of these diseases increased to 86% in females and 82% in males (2012 NHLBI Fact Book) [20]. Recently published data have shown that these diseases are the leading cause of death for individuals aged >65 [21] and morbidity increased from 32% for individuals aged 66 to 48% for individuals aged 85. An important factor which significantly decreases the incidence of coronary heart diseases in postmenopausal women is estrogen [22–24]. In women already having coronary artery disease or ischemic stroke, the therapeutic benefit of estrogen is not clear [25, 26] although it has been reported that estrogen induces rapid vasodilation, exerts anti-inflammatory activity, and regulates vascular cell growth, migration, and protection of cardiomyocytes from injury [27], all of which prevent atherosclerotic deterioration in vessels. This review focuses on the role of EMPs in angiogenesis, coagulation, and inflammation during age-related vascular diseases and the contribution of estrogen to these diseases. 

## 2. Endothelial Microparticles and Factors That Stimulate Their Formation and Release

EMPs are small vesicles that are released from endothelial cells and can be found circulating in the blood. Defined by their small size (0.1 to 1.0 *μ*m), they are a heterogeneous population of vesicles which are shed from plasma membranes in response to cell activation, injury, angiogenesis/neovascularization, and/or apoptosis. EMPs consist of a small amount of cytosol surrounded by a plasma membrane and display negatively charged phospholipids on their surface that can initiate and accelerate coagulation [28]. Circulating EMPs have been demonstrated as a marker of preeclampsia [29, 30], acute coronary syndromes [31], and severe hypertension [30, 32, 33] suggesting their association with pathological processes within the endothelium [34]. Furthermore, circulating EMP levels are also implicated in the progression of atherosclerotic lesions, heart failure, arrhythmias, inflammatory vascular disease, sickle anemia, and endotoxemia [35]. 

**Figure 1:**
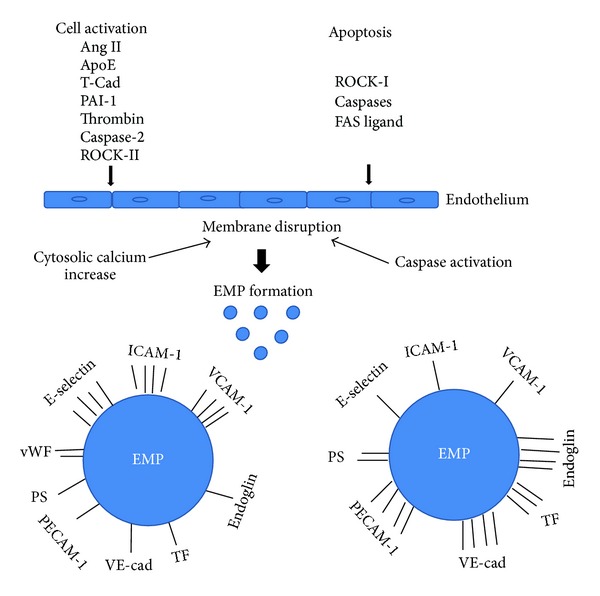
Differences in antigen expression of EMPs derived from activation versus apoptosis of endothelial cells. Endothelial cell activators, angiotensin II (Ang II), apoliprotein E (ApoE), T-cadherin (T-Cad), plasminogen activator inhibitor-1 (PAI-1), and thrombin, cause cytosolic calcium increase which leads to endothelial cell membrane disruption. Caspase-2 activates ROCK-II independently of cell death [45]. Apoptosis inducers including Rho activated kinase (ROCK-I), caspases, and FAS ligand activate caspases and cause membrane disruption in endothelial cells. Both activators of cell activation and apoptosis lead to vesiculation and EMP generation. Bars represent level of antigen on the surface of the EMP. 4 bars: high level of antigen; 1 bar: low level of antigen. EMP antigens: E-selectin, intracellular adhesion molecule-1 (ICAM-1), vascular cell adhesion molecule-1 (VCAM-1), platelet cell adhesion molecule-1 (PECAM-1), endoglin, vascular endothelial-cadherin (VE-cadherin), tissue factor (TF), phospholipid (PS), and von Willebrand factor (vWf). This figure was prepared based on [13, 37–40, 42, 45].

EMPs can display differing characteristics that are reflective of specific thrombotic and inflammatory conditions [36]. Jimenez et al. demonstrated that the surface antigens of EMPs are distinctive depending on the type of endothelial cell injury, as in apoptosis *versus* activation. High levels of the surface antigens E-selectin, intracellular adhesion molecule-1 (ICAM-1), and vascular cell adhesion molecule-1 (VCAM-1) are on EMPs derived from activated endothelial cells. In contrast, the low levels of these antigens are on EMPs derived from apoptotic endothelial cells. Platelet cell adhesion molecule (PECAM-1), endoglin, and vascular endothelial-cadherin (VE-cadherin) are at low levels on EMPs derived from activated ECs [37–40] (Figure 1). Additionally, EMPs generated from apoptotic endothelial cells have higher levels of phosphatidylserine on their surface and different phospholipid composition and oxidation status compared with EMPs generated from activated endothelial cells [41, 42]. These data suggest that there are distinct mechanisms for the formation of EMPs in apoptotic and activated cells [43] and several studies suggest that these types of EMPs have different functions in vascular diseases [40, 44].

**Figure 2:**
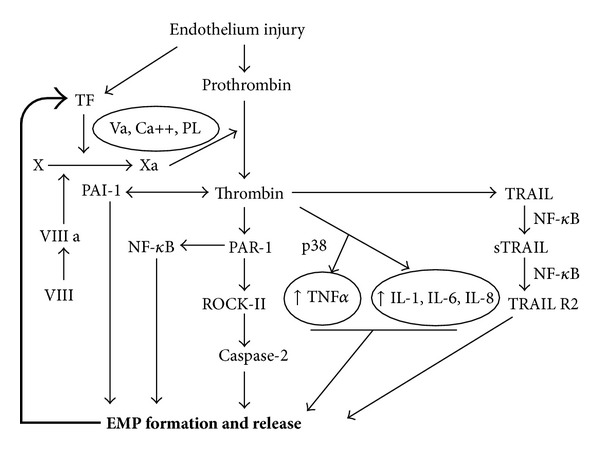
Signaling pathways involved in thrombin induced EMP formation. EMPs carry TF, the main initiator of the extrinsic pathway of the coagulation cascade. Cofactor VIIIa known as a von Willebrand factor activates factor X to Xa in the presence of factor Va, calcium, and phospholipids (PL), which results in the generation of thrombin. Thrombin induces tumor necrosis factor alpha (TNF*α*) via p38 (mitogen activated protein kinase) and interleukins 1, 6, and 8 (Il-1, IL-6, and IL-8), which both lead to formation of EMPs. Thrombin, via proteolytically activated receptor-1 (PAR-1), induces nuclear factor kappa B (NF-*κ*B), which directly induces EMP formation. PAR-1 also induces Rho kinase (ROCK- II) which activates caspase-2 leading to EMPs formation. Thrombin via (tumor necrosis factor related apoptosis inducing ligand) TRAIL activates sTRAIL, which is synthesized to TRAIL R2. This process requires the activation of NF-*κ*B.

Both inflammatory cytokines and coagulation factors participate in the generation of EMPs (Figure 2).

Recently, it has been shown that p38 mitogen-activated protein kinase (MAPK) is a critical molecule in the production of proinflammatory EMPs and increased ICAM-1 production by endothelial cells, providing a paracrine loop to enhance the endothelial response to inflammation [46]. *In vitro* studies have shown that the proinflammatory agent, TNF*α*, activates endothelial cells and induces release of EMPs [47] (Figure 2). Another potent stimulus for EMP formation both *in vivo* and *in vitro* is angiotensin II (Ang II) [3]. This effect is mediated by Ang II receptor type I that signals through NADPH oxidase and Rho kinase. Furthermore, in Ang II-infused apoliprotein E (ApoE −/−) hyperlipidemic mice, a model of significant endothelial dysfunction, Ang II has been shown to increase EMP formation by a redox-sensitive and blood-pressure-independent process [2].

**Figure 3:**
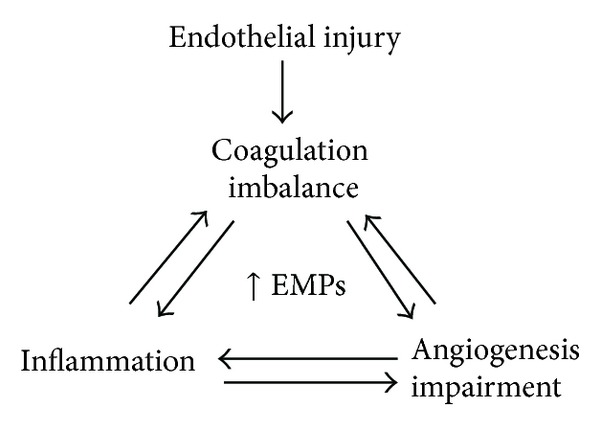
EMPs link inflammation, coagulation, and angiogenesis.

Another important factor PAI-1 (plasminogen activator inhibitor type 1) plays an important role in the formation of EMPs. It has been shown by Brodsky et al. that PAI-1 promotes formation of EMPs with reduced transmembrane asymmetry of phospholipids in a dose dependent manner. This occurrence may be responsible for the observed increase in *in vitro* thrombin generation. These findings could possibly link elevated levels of PAI-1 with endothelial dysfunction and tendency toward thrombosis [48–50]. Increased levels of PAI-1 might serve as an initiator of EMP formation followed by increased procoagulant activity and thrombin generation [50]. In addition, it is also known that thrombin stimulates PAI-1 synthesis [51] suggesting constant production of these two factors. All these data indicate that formation of EMPs links together inflammation, coagulation, and angiogenesis and causes the impairment of the last two phenomena (Figure 3).

Among many signaling molecules, T-cadherin (T-cad) on the surface of ECs might be upregulated and may serve as a characteristic marker of EC activation and stress. Recently, Philippova et al. have demonstrated a mechanism of T-cad-dependent signaling in the vascular endothelium. The authors identified that T-cadherin levels in plasma are increased in early atherosclerosis and correlate with endothelial dysfunction, which may lead to increased release of EMPs from ECs [52].

## 3. Endothelial Microparticles in Coagulation and Vascular Aging

Increasing evidence has also accumulated to implicate an impaired coagulation system in many vascular diseases. This coagulation imbalance is the net result of activation of coagulation, impaired activity of natural coagulation inhibitors, and suppressed fibrinolysis [53]. Activation of coagulation proteases, for example, thrombin, is one of the earliest events following tissue injury [54]. Thrombin modulates tissue repair responses by altering vascular permeability, stimulating endothelial cell, fibroblast, and neutrophil migration, and promoting their spreading and adhesion [55]. It activates various cell types and induces secretion of several proimmune, profibrotic, and proinflammatory factors [45, 56, 57]. Additionally, thrombin induces generation of EMPs. Recent studies by Sapet et al. have shown that release of EMPs by endothelial cells in response to thrombin involves a group of genes that regulate angiogenesis and are linked to the cytoskeleton reorganization family. Among these genes, Rho-kinase ROCK-II was transcribed at a high rate and was identified as a target of thrombin in EMP generation [58]. The involvement of caspase-2 in ROCK-II activation, independent of cell death, points out a novel signaling pathway that emphasizes the proteolytic activity of caspase in EMP generation in response to cell activation [58] (Figure 1). However, further studies are needed to determine the molecular mechanisms involved in EMP release. EMP release is initiated when thrombin binds to its receptor, proteolytically activated receptor-1 (PAR-1), which induces gene transcription that is mediated by thrombin via TRAIL/Apo2L, a cytokine belonging to the tumor necrosis factor alpha (TNF*α*) superfamily. This mechanism of EMP generation depends on the nuclear factor (NF) *κ*B activation and involves the soluble form of TRAIL, which is secreted by the endothelial cells under thrombin or inflammatory stimulation [4, 59] (Figure 2).

Phospholipids expressed on EMPs bind coagulation factors leading to a prothrombotic state [60] and an increase in procoagulant activity of tissue factor (TF). These phospholipids are exposed on the outer membrane of MP and are considered to be the main initiators of the coagulation cascade [28]. Additionally, it has been demonstrated that sphingosine 1-phosphate (S1P) strongly potentiates thrombin-induced TF expression in ECs suggesting its role in blood coagulation [61]. S1P has also been shown to be involved in the process of angiogenesis and inflammation [62–64]. Another important role of MPs is their contribution to the development of platelet- and fibrin-rich thrombi at sites of vascular injury via the recruitment of cells and the accumulation of TF. Data suggest that EMP-mediated coagulation has clinical significance; for example, an association between the number of circulating MPs and the risk of thrombolytic complication has been reported [19]. Because EMPs interact with coagulation proteins and with inflammatory or vascular cells, their role in cardiovascular diseases has been intensively studied. It has also been observed by Jy et al. that EMPs carry von Willebrand factor (vWf) and factor VIII that promote platelet aggregates and increase their stability [65] (Figure 1). Moreover, the authors postulated that EMPs released during vascular injury may arrest bleeding by rapid interaction with platelets via membrane-associated vWf multimers and adhesions to stabilized platelet aggregates in the microenvironment. Sabatier demonstrated that EMPs also carry TF and bind to monocytes causing further TF expression and resulting in enhanced transmigration of monocytes through endothelial junction [66, 67]. 

**Figure 4:**
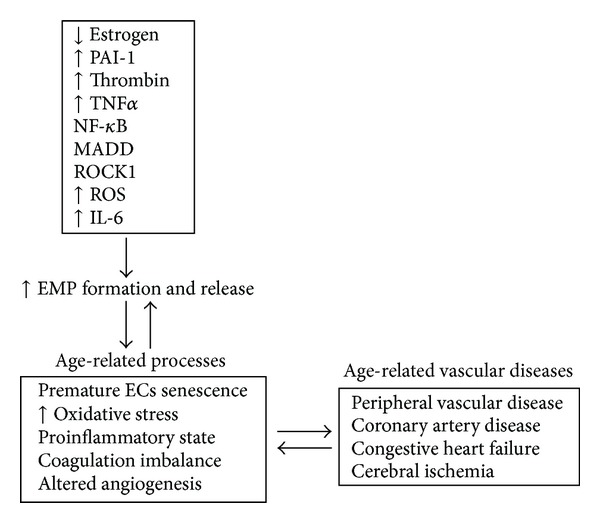
Factors contributing to EMP formation in age-related vascular diseases.

One of the important key genes for aging-associated cardiovascular disorders is plasminogen activator inhibitor-1 (PAI-1), a main inhibitor of fibrinolysis. The expression of PAI-1 is not only elevated in the elderly but also significantly induced in a variety of pathologies associated with the process of aging [68]. Increased levels of PAI-1 and its procoagulant activity have been recognized as hallmarks of endothelial dysfunction in vascular aging (Figure 4). Furthermore, elevated levels of PAI-1 were found in Werner syndrome, a disease characterized by premature aging [68, 69] and atherosclerosis, which in advanced stages may lead to myocardial infarction and death. Recently, it has been shown that EMPs expressing both activators and inhibitors of coagulation have fibrinolytic properties that counteract their procoagulant activities, which may enable them to contribute to haemostatic balance [70]. It has been observed that endothelial and leukocyte microparticles generate fibrinolytic activity, whereas erythrocyte and platelet microparticles do not have this property. Additionally, different plasminogen activators were identified on leukocyte microparticles, urokinase-type plasminogen activator (uPA), and EMPs where tissue plasminogen activator (tPA) has been found. The authors provide evidence that microparticles with plasminogen activators are rare in healthy populations but are observed more frequently in pathological conditions [70] suggesting that plasmin generation on microparticles may be important in the modulation of hemostatic balance. Therefore, complex functions of EMPs have an ambivalent role both in physiological and pathological conditions, either promoting or inhibiting coagulation, inflammation, or angiogenesis. However, the precise mechanism has not yet been explored.

## 4. Endothelial Microparticles in Inflammation

There is increasing evidence that inflammation is a potent activator of coagulation pathways. Inflammatory mediators increase several procoagulant factors, inhibit endogenous anticoagulants, and attenuate the fibrinolytic response [71]. However, the interaction between these two systems is bidirectional, as coagulation is also capable of modulating inflammatory activity. Thrombin mediated inflammatory molecules such as IL-8 and IL-1Ra and IL-1 participate in EMP release [4, 59] (Figure 2) and are key factors involved in coagulation, inflammation, and angiogenesis. Inflammation and coagulation are linked processes in many diseases and EMPs may amplify the responses by activating the endothelium. It has been reported that in addition to activation of D-dimers and C-reactive proteins in coagulation, the inflammatory cytokine IL-6 is associated with mortality, declines in all measures of function, and leads to the frailty phenotype in the elderly [72]. Recently, it has been shown that increased levels of IL-6 are present in aged aortas and that aging induces a proinflammatory phenotype in vascular smooth muscle cells (VSMC) due in part to increased signaling of toll-like receptor 4 (TLR4) and its signaling adaptor MYD 88 [73]. These observations support the notion of a high prevalence of proinflammatory conditions in advanced age. All of these factors lead to increased generation of EMPs and impaired coagulation, inflammation, and angiogenesis in CV diseases (Figures 3 and 4).

## 5. Endothelial Microparticles and Their Effect on Vascular Function in the Elderly

The process of angiogenesis is complex and requires endothelial cells (ECs) to detach from pericytes and the extracellular matrix (ECM), proliferate, invade the surrounding tissues, migrate, and differentiate to form capillary tubes that connect to newly developed vascular networks leading to vascular stabilization [74–76]. Defective angiogenesis has been found in many vascular diseases and it has been established that EMPs play an important role in this process. MPs can act on angiogenesis directly through ligand/receptor interaction or indirectly by modulating production of soluble factors involved in endothelial cell differentiation, proliferation, migration, and adhesion [62]. Brodsky et al. have shown that in low concentration, EMPs did not affect the endothelium. However, increased levels of circulating EMPs are an important factor in the pathophysiology of CV diseases, directly affecting the endothelium and other circulating cells [77]. One of the mechanisms mediating these changes may be increased oxidative stress. Brodsky et al. have demonstrated that EMPs directly impair vasorelaxation via diminishing production and/or bioavailability of nitric oxide. This result was correlated with increased superoxide levels in aortic rings isolated from rats and cultured endothelial cells treated with microparticles [77]. Other studies have demonstrated that MPs of endothelial origin induce the expression of endothelial cyclooxygenase type 2, different adhesion molecules, release of cytokines, and impaired release of nitric oxide from vascular endothelial cells [78]. High EMP levels may be considered a biomarker of vascular damage [79]. A pathological concentration (≥10^5^) of EMPs affects angiogenesis by diminishing the cell proliferation rate and decreasing the total capillary length of human umbilical vein endothelial cells (HUVEC) plated on a matrigel substrate [80]. Moreover, HUVECs or human microvascular endothelial cells (HMVECs) treated with EMPs demonstrated disorganized tube formation in the presence or absence of VEGF [80]. This data suggests that EMPs regulate EC function and disrupt growth factor signaling. Many aspects of endothelial cell function and angiogenic capacity are also altered with age. Impairments in the regulation of vascular tone, coagulation, and hemostasis contribute to damage of the vascular system and dysfunctional angiogenesis [81–83]. Recently, *in vitro* studies published by Burger et al. have proved that a long term culture of mouse aortic ECs leads to a senescent phenotype with increased ROCK activity and formation of MPs (Figure 4). Furthermore, it has been shown that MPs promote premature EC senescence through the stimulation of endothelial cell ROS production [84] (Figure 4). Brodsky et al. demonstrated a significant increase in the number of circulating EMPs in obesity-induced diabetes rats as well as premature endothelial cell senescence and vasculopathy. This disorder was characterized by impaired vasorelaxation, nitric oxide production, and defective angiogenesis. An increased number of circulating EMPs have been identified in patients with certain diseases, such as hypertension, coronary artery disease, acute coronary syndrome, and stroke [81]. In patients with established endothelial dysfunction, levels of circulating EMPs are inversely correlated with the amplitude of flow-mediated dilation, independent of blood pressure [11, 85–87]. Another study published by Thomasow et al. has shown elevated levels of CD31^+^ (PECAM-1), which are suggestive of EC apoptosis, in severe and mild chronic obstructive pulmonary disease (COPD) and emphysema. CD31^+^ EMPs were positively related to emphysema and were inversely associated with pulmonary microvascular blood flow. In contrast, CD62E^+^ (E-selectin) EMPs indicative of endothelial activation were elevated in severe COPD and hyperinflation. These cellular markers may involve endothelial apoptosis in the pathogenesis of emphysema and COPD [88]. Previous studies have demonstrated an impairment of cell proliferation, migration, tube formation, and sprouting in older individuals (>65 years, male or female) when compared to their younger counterparts (<65 years, male or female) suggesting that these changes contribute to the decrease in effective blood vessel growth and repair mechanisms in the elderly [89]. A decrease of proangiogenic factors and loss of circulating endothelial progenitor cells (EPCs) have also been observed with increased aging (>50 years, male), which may lead to compromised angiogenesis [90]. Furthermore, age-related changes in EPC number and function may directly correlate with the degree of senescent endothelial impairment [91]. Furthermore, in older men (>60 years) with myocardial infarction, circulating microparticles selectively impair the nitric oxide transduction pathway in endothelial cells, which contributes to the general vasomotor dysfunction observed after myocardial infarction [33]. Additionally, an environmental alteration such as a decrease in ECM proteins particularly fibrillar collagen production and small leucine rich proteoglycans (SLRPs) [92] and changes in the expression of matrix metalloproteinases (MMPs) have also been observed with increased aging [93]. EMPs are clearly implicated in the impairment of angiogenesis in vascular diseases associated with aging however, the specific pathways through which EMPs augment this process are unknown. Moreover, EMPs may be one of the major factors leading to reduced effectiveness of therapies for treating impaired angiogenesis in humans and should be further explored. 

## 6. Endothelial Microparticles and Estrogen in Age-Related Vascular Diseases

Aging and estrogen loss are strongly linked. Furthermore, estrogen levels have been connected to thrombin generation, a central molecule in the coagulation cascade in postmenopausal women [94]. Estrogen plays an extraordinary role in normal vascular development and vascular diseases. It has been shown that estrogen directly modulates angiogenesis via multiple pathways. In particular, estradiol increases migration, proliferation, and formation of capillary-like networks of HUVECs [95] by the classic estrogen receptor (ER) pathway [96–98]. Most reports have concentrated on the role of ERs in mediating big vessel relaxation and contraction. In a rat model of acute myocardial infarction it has been shown that estradiol promotes myocardial angiogenesis by increasing microvascular density through estrogen receptors [99]. Furthermore, in ovariectomized rats it has been demonstrated that an increased number of genes in the aged heart, including TNF and MAP kinase-activating death domain protein (MADD), play a role in the release of EMPs [100–102] (Figure 4). Another *in vivo* study has shown that ovariectomy in female rats is associated with reduced PAI-1 expression, while estrogen replacement counteracts this change promoting EMP formation [103]. *In vitro* studies have demonstrated that estrogen induced PAI-1 expression is implicated in HUVEC horizontal migration [103]. Other mechanisms have also been revealed to be involved in the proangiogenic effect of estrogen including increased expression of both VEGF and its receptors [104, 105] and bFGF [106], as well as expression of vascular adhesion molecules [107]. Moreover, estrogen is known to enhance nitric oxide production and release by endothelial cells [108]. Studies published by Reed and Edelberg have suggested that a physiological decrease in the concentration of steroid hormones (e.g., estrogen and testosterone) as a result of menopause and “chronological aging” may contribute both directly and indirectly to subsequent deficits in the synthesis and function of the angiogenic growth factor TGF-*β* [109]. 

In humans endogenous estrogen contributes to the anticoagulant, anti-inflammatory, and antithrombotic properties of the endothelium. The numbers of endothelium, platelet, and monocyte-derived microparticles have been found to be elevated in low-estrogen menopausal women. The authors implied that increased numbers of procoagulant microparticles provide a resource to study mechanisms for cardiovascular risk development in newly menopausal women [110]. On the other hand, studies demonstrated by Rank et al. [111] had shown that hormone replacement therapy in postmenopausal women increased the concentration of MPs derived from platelets. The EMP levels were unchanged excluding their primary role in the initiation of a thromboembolic event in these women [112]. Another study has shown that EMP concentration was diminished in older patients (>80 years, male or female) but MP procoagulant activity was preserved [113] in comparison to younger patients. The older patients had higher incidence of hypertension and stable coronary disease. The authors suggested that a decreased EMP level was associated with age and any effect of gender was ruled out by multivariate analysis. The study performed by Mateos-Caceres [78] had shown an increased level of circulating EMPs in elderly (>66 years) male and female patients with acute stroke and that TNF activated EMPs were the major player in stroke induction. Furthermore, Simak et al. demonstrated that circulating EMP phenotypes may be associated with the severity, lesion volume, and outcome of acute ischemic stroke (AIS) in male patients (>78 years) [113]. On the other hand, studies published by Williams et al. have shown that in elderly patients of both genders (>66 years), EMP levels were similar in AIS and stroke mimic patients [114]. Moreover, they demonstrated that EMPs were generated via activation and not by apoptosis/necrosis of endothelial cells. This suggested that EMPs may not be an appropriate marker for AIS, given the incapability to distinguish between AIS and stroke mimic. The tree-city cohort studies have shown that increased thrombin generation is an independent predictor of AIS in elderly women suggesting that hypercoagulability may play an important role in the pathogenesis of AIS [115]. Furthermore, the population-based cohort studies published by Hoekstra et al. indicated that elevated plasma PAI-1 levels are a strong risk factor for stroke at old age in people of both genders; however, the 4G/5G polymorphism variant of PAI-1 is associated with reduced incidence of stroke [116]. Furthermore, PAI-1 has been suggested as a gene predisposing peripheral hypertension. It has been shown that the single polymorphism PAI-1 4G/5G genotype is associated with higher central systolic, diastolic, and mean arterial blood pressure in women (>70 years), while no association was found in men [117] suggesting gender specific biology of PAI-1 in addition to an advanced age specific factor. Moreover, elevated levels of circulating MPs have been reported in patients (>58 years, male or female) with acute myocardial infarction and coronary artery disease [118]. Studies published by Sinning et al. [119] have shown that circulating EMPs, but not MPs of other cellular origin, are a strong predictor of cardiovascular mortality and major cardiovascular events in patients (>66 years, male or female) with coronary artery disease and pulmonary hypertension [120]. All these data may indicate that estrogen probably does not exert its protective effects on CV diseases through the EMP axis. However, more analyses are needed in order to confirm if a direct connection occurs between estrogen and EMPs in age-related vascular diseases (Figure 4). 

## 7. Conclusions

Endothelial microparticles, as pleiotropic factors, play a role in both physiological and pathological conditions and thus may contribute to regulation of vascular homeostasis. EMPs not only reflect the stage of disease but also play a causative role in the development of various vascular diseases. They can modulate coagulation, and their elevated levels have been observed in many conditions associated with inflammation and angiogenesis. The prothrombotic properties and proinflammatory effects of microparticles on endothelial cells affect vascular aging and lead to structural changes in the heart and other organs. Thrombin and PAI-1 seem to be key factors involved in EMP generation in age-related vascular disease. Furthermore, in age-related vascular diseases, steroid hormones are among the factors that have been shown to have an influence on vascular homeostasis. Specifically, estrogen plays a regulatory function on vessel inflammation, injury, and repair. Lack of estrogen has been suggested to be directly involved in the endothelial cell injury with EMP release that is observed in ischemic diseases. However, the direct link between EMP generation, EMP release, and estrogen is understudied and the further investigation of cellular and molecular mechanisms of these correlations is imperative for understanding and providing a basis for new translational investigations. Furthermore, circulating EMPs show great promise not only as biomarkers in the diagnostics of vascular diseases but also as a target for the treatment of these disorders, especially in elderly patients.
